# Habitat Occupancy of the Critically Endangered Chinese Pangolin (*Manis pentadactyla*) Under Human Disturbance in an Urban Environment: Implications for Conservation

**DOI:** 10.1002/ece3.70726

**Published:** 2024-12-15

**Authors:** Asmit Subba, Ganesh Tamang, Sony Lama, Jash Hang Limbu, Nabin Basnet, Randall C. Kyes, Laxman Khanal

**Affiliations:** ^1^ Central Department of Zoology, Institute of Science and Technology Tribhuvan University Kathmandu Nepal; ^2^ Nature Conservation and Study Center Kathmandu Nepal; ^3^ Central Campus of Technology Tribhuvan University Dharan Nepal; ^4^ School of Ecology and Nature Conservation Beijing Forestry University Beijing China; ^5^ College of Fisheries and Life Science Shanghai Ocean University Shanghai China; ^6^ Central Department of Botany, Institute of Science and Technology Tribhuvan University Kathmandu Nepal; ^7^ Departments of Psychology, Global Health, and Anthropology, Center for Global Field Study, and Washington National Primate Research Center University of Washington Seattle Washington USA

**Keywords:** anthropogenic threats, Chinese pangolin, occupancy, termite mounds, urban landscape

## Abstract

Globally, urban expansion has led to habitat fragmentation and altered resource availability, thus posing significant challenges for wildlife. The Chinese pangolin ( *Manis pentadactyla* ) is a critically endangered species experiencing population decline due to illegal trade and habitat degradation. This study analyzed variables affecting habitat occupancy of Chinese pangolins using a single‐season occupancy model across 134 study grids (600 m × 600 m) in peri‐urban areas of Dharan Sub‐Metropolitan City, eastern Nepal. We identified termite mounds as a significant key factor (top model with AICwt = 1) in the detection probability of Chinese pangolin burrows (*β*_Termite mounds_ = 1.48, 95% CI = 1.07 to 1.89). Additionally, the Human Disturbance Index (HDI) emerged as the key variable for habitat use occupancy (AIC = 231.96, AICwt = 0.309), indicating a significant negative impact (*β*_HDI_ = −6.555, 95% CI = −11.324 to −1.7723). We observed a mean HDI of 0.475 ± 0.04 in the grids where Chinese pangolins were detected, with higher HDI values correlating with reduced Chinese pangolin occupancy. For the long‐term conservation of Chinese pangolins in urban landscapes, it is crucial to reduce anthropogenic activities and implement conservation measures to protect suitable habitats with abundant termite mounds.

## Graphical abstract

This study analyzed detection probability and habitat occupancy of Chinese Pangolin using single‐season occupancy modeling. The number of termites mounds increases detection probability, while Human Disturbance Index (HDI) decreases the habitat occupancy. The findings emphasize the need for targeted conservation efforts, considering the fine‐scale ecological and anthropogenic factors impacting Chinese pangolins in urban and peri‐urban areas.

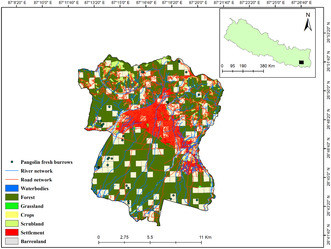

## 1 Introduction

Globally, urban areas are expanding, resulting in a change from rural to urban life for more than 55% of the world's human population. This trend is expected to increase to 68% by 2050 (United Nations 2018). In urban areas, a large percentage of land has been converted to human use, resulting in habitat fragmentation, reduced structural complexity, and altered resource availability. The response of mammalian species to an urban landscape is often species‐specific, with some showing neutral or positive responses and others displaying negative responses to the modified landscape (Brady et al. 2011; Schuttler et al. 2021). Studies have shown that the nature of the response depends in part on the animals' body size (Brady et al. 2011; Goad et al. 2014; Nicholson and Van Manen 2009), temporal activity and predator–prey relations (Fardell et al. 2022), and both intraspecific and interspecific interactions (Brady et al. 2011; Prevedello et al. 2013). For some mammals living in human‐modified landscapes, their population densities have fared positively due to a combination of species filtering, increased resources, possible reduction in competition and predation, and having an evolutionary potential to change (Caspi et al. 2022; Tucker et al. 2021). Other species, however, especially those that tend to be more specialized, often experience a great negative impact in an urban landscape due to habitat modification, alterations in food dynamics, human‐induced road kills, and so forth (Fitzgibbon, Wilson, and Goldizen 2011; Goad et al. 2014; Nicholson and Van Manen 2009).

The Chinese pangolin ( *Manis pentadactyla* ) is listed as critically endangered in the IUCN Red List of Threatened Species (Challender et al. 2019) and is reported to be the world's most trafficked mammal species (Challender et al. 2019; Zhang et al. 2017). They have a myrmecophagous food specialization and depend largely on specific ants and termites for their diet (Tamang, Sharma, and Belant 2022). Further, they have limited defense mechanisms (Spearman 1967), which makes them more vulnerable to extinction. They inhabit a variety of habitats throughout their range, including bamboo forests, grasslands, and agricultural fields and primary and secondary forest in peri‐urban areas (Bao et al. 2013; Lee et al. 2017). Anthropogenic threats such as forest fires, hunting, trade (Challender et al. 2019), seasonal climate variations, slope and aspect (Shrestha et al. 2021), canopy cover (Katuwal, Sharma, and Parajuli 2017), soil moisture, ground texture, and the availability of food resources like ants and termites (Cornelius and Osbrink 2010) are interconnected variables that influence their habitat and occupancy (Wu et al. 2003).

In Nepal, 94% of potential Chinese pangolin habitat is found outside of protected areas in human‐modified landscapes (e.g., cultivated lands, human settlements, and infrastructure development areas). Of this total suitable habitat, 44% lies in the mid‐hills (1000–3000 m asl) (Sharma, Rimal, et al. 2020, Sharma, Sharma, et al. 2020). In contrast, the Siwalik and Tarai plain areas (below 1000 m asl) in eastern and central Nepal, the lowest elevation within their range, show the least suitability, accounting for only 14% of the total suitable habitats (Sharma, Rimal, et al. 2020, Sharma, Sharma, et al. 2020). This reduced suitability is primarily driven by the ongoing conversion of former forests into agricultural land and settlements (Paudel et al. 2016) and is further compounded by the high level of urbanization and human activity (Rimal et al. 2019). The loss of forest cover between 2001 and 2021 resulted in an annual emission of 497 tons of CO_2_, primarily due to deforestation and forest fires (GFW 2022). This loss has severely impacted the Chinese pangolins' primary habitat. Despite these significant threats, the status of Chinese pangolins and the factors affecting them in urbanized areas of the Siwalik and Tarai regions remain under‐studied. This lack of information hampers the development of effective conservation strategies and management plans for the species in these critical areas.

Considering their threatened status, specialized dietary habits, and higher anthropogenic threats in changing urban landscape, we conducted this study in an effort to fill in the information gap on the Chinese pangolin inhabiting the urban habitat of Dharan Sub‐Metropolitan City of eastern Nepal. It is essential to investigate how the Chinese pangolins use habitat patches of high anthropogenic activity in an urban landscape so that we can formulate effective local‐level management and conservation plans. We hypothesized that anthropogenic activities negatively impact the habitat occupancy of Chinese pangolins. We used single‐species‐single‐season occupancy modeling based on sign surveys to identify the patterns and determinants of habitat occupancy by Chinese pangolins under anthropogenic disturbances in an urban landscape of eastern Nepal.

## 2 Materials and Methods

### 2.1 Study Area

**FIGURE 1:**
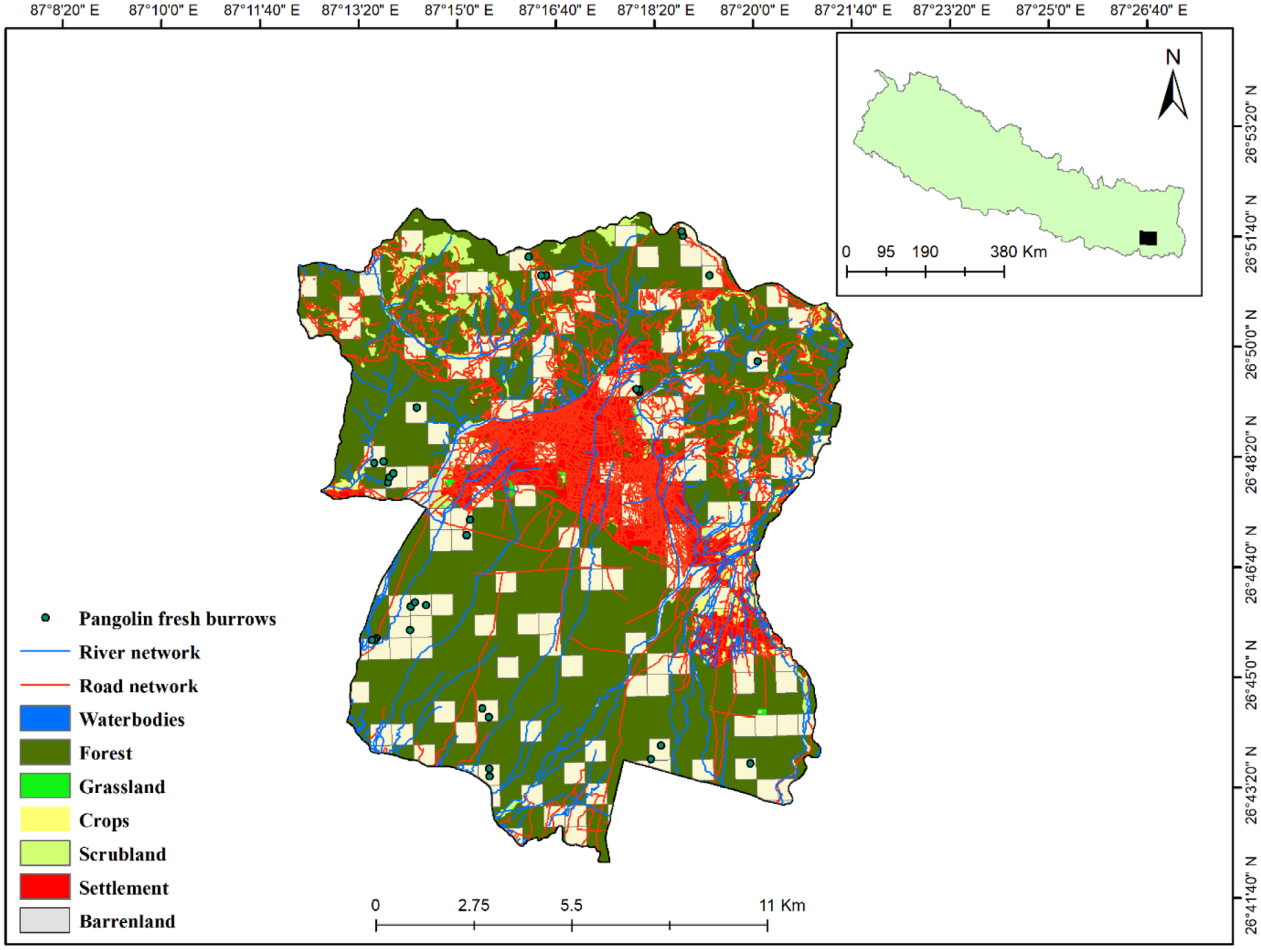
A map depicting Dharan Sub‐Metropolitan City study area and survey locations. Beige study grids (600 × 600 m) indicate survey sites, blue lines represent rivers, and red lines represent road networks.

Dharan Sub‐Metropolitan City (Dharan SMC) lies in the Sunsari District of Koshi Province in eastern Nepal with its southern tip sharing the boundary with the lowland Tarai area (Figure 1). The city covers an area of 192.2 km^2^ and ranges in elevation from 119 m asl to 1778 m asl on the northern foothills of the Siwalik Range. There are seven land cover types in Dharan: water, forests, grasses, crops, shrubs, built areas, and barren grounds. The majority of the Dharan region (ca. 151 km^2^) is covered by forest (Ghimire, Higaki, and Bhattarai 2013). The predominant vegetation type is *Shorea robusta*, complemented by tropical evergreen tree species and Himalayan subtropical broadleaved forests. The Terai‐Duar savanna and tropical and subtropical grasslands and shrublands also make up the Dharan area. A moderate and temperate climate with a cold and dry winter and hot and humid summer predominates throughout the region (Ghimire, Higaki, and Bhattarai 2013).

Two major streams, the Sardu and Seuti, border Dharan on the west and east sides, respectively. Several minor streams, including the Khahare Khola (Nepali word for a stream) and Turke Khola, pass through Dharan (Aksha et al. 2020). However, only 0.45 km^2^ of Dharan is covered by aquatic habitat. Followed by the forest, the built‐up area makes up the second‐highest coverage, as determined by analysis using Sentinel‐2 data. Dharan lost 18 ha of tree cover between 2001 and 2021, which is equivalent to a 0.17% decline since 2000. Another 0.826 ha of tree cover was lost due to forest fire and 17 ha due to all other factors, including illegal logging, haphazard resource extraction, and unsustainable developmental projects (GFW 2022).

### 2.2 Sampling Design and Field Data Collection

The single‐season occupancy framework was used to identify the Chinese pangolin distribution in the urban landscape characterized by significant human‐induced threats to the species. With the help of Create Fishnet tools in Arc GIS 10.5, the study area was divided into multiple grids (600 m × 600 m) following standard methods for pangolin monitoring (DNPWC 2019). The grid system, generated using the clip analysis tool, resulted in a total of 609 grids. A subset of the grids, referred to as study grids, was randomly chosen using the Subset Feature Class tools in ArcGIS 10.5. A total of 152 study grids were selected for the survey which covered 25% of the total study area. However, 18 of the grids were excluded from the survey due to challenging terrain and because they covered less than 50% of their total area within the survey boundary. This left a total of 134 study grids targeted for survey. In each study grid, a continuous belt transect of 600 m length and 50 m width was surveyed. The survey data collected along each transect were partitioned into 100 m segments along each transect for the analysis (Mackenzie and Royle 2005). Each grid was surveyed once from 16 November 2020 to 24 February 2021 by a team of three skilled surveyors. On average, each grid took 2 h survey, and all surveys were conducted between 08:00 and 16:00 h.

Chinese pangolins are ground dwelling, nocturnal, and a highly elusive species. Therefore, we relied on a widely accepted indirect sign survey approach based on sightings of fresh burrows as indication of Chinese pangolin presence in the site (Ingram et al. 2018; Willcox et al. 2019). Burrows with fresh, loose soil with fresh pugmarks and scats near the entrance were defined as active burrows and grids with such burrows were considered as presence grids (DNPWC 2019). Chinese pangolin shows sympatric overlaps with the Indian Crested porcupine ( *Hystrix indica* ) in the mid‐hills of Nepal (Jnawali et al. 2011). Therefore, burrows identified with a single entrance, having a semicircular shape, and featuring a heap of soil at the entrance, were classified as Chinese pangolin burrows (DNPWC 2019). Thirteen burrows observed within the study area belonging to the Indian crested porcupine were excluded from the analysis. During the transect surveys, we collected Chinese pangolin detection/non‐detection data as well as field‐based covariates (described below) grouping for each 100 m segment. Occupancy data were created from each study grid, with fresh burrows recorded as “1” to indicate detected grids and as “0” for grids where no burrows were detected (Sharma, Rimal, et al. 2020, Sharma, Sharma, et al. 2020). Old burrows were excluded from the analysis. Detection histories were then constructed by aggregating the segment‐level detection/non detection data, whereas the values of the field‐based covariates were averaged from each grid to form site covariates (Pokharel et al. 2022).

### 2.3 Environmental Covariates

Based on a comprehensive literature review of factors influencing distribution and ecology, we identified key variables that are likely influential in shaping the spatial patterns of Chinese pangolins in our study area, namely, anthropogenic threats (HDI), food availability (Termite mounds), topographic heterogeneity, Normalized Difference Vegetation Index (NDVI), and distance to water (Table 1). Factors such as forest fires (both active and inactive, identified through charcoal and burned vegetation), livestock grazing, nearest settlements, and nearest foot trails are found to have a negative association with the occurrence of Chinese pangolins suggesting that pangolins tend to avoid areas with significant anthropogenic activities (Katuwal, Sharma, and Parajuli 2017). Chinese pangolins also exhibit food specialization and selectivity, often consuming specific ants and termites (Lee et al. 2017; Wu et al. 2005). Additionally, their foraging behavior is influenced by the distance to the ant and termite mounds/nests (Tamang, Sharma, and Belant 2022). Similarly, the detection of Chinese pangolins in Nepal is influenced by topography characterized by diverse habitats, including areas with moderate to high canopy coverage, forest and agricultural land, and proximity to nearest water sources (Katuwal, Sharma, and Parajuli 2017; Tamang, Sharma, and Belant 2022). Based on other studies, we hypothesized that the presence and number of termite mounds, distance to nearest water source, NDVI, and topographic heterogeneity would have positive influence on the occupancy of Chinese pangolins in the study area. Additionally, we predicted that anthropogenic activities would exert a negative influence on Chinese pangolin occupancy.

**TABLE 1:** Covariates used to test their influence on the habitat use of Chinese pangolins in the urban landscape of Dharan Sub‐Metropolitan City.

Covariate	Prior prediction direction of influence	All sampling sites		Sign detection sites	
		Mean	SE	Mean	SE
Human disturbance index (HDI)	Negative	0.74	0.0197	0.475	0.0401
Termite mounds	Positive	0.9408	0.1983	2.55	0.1983
Distance from the nearest water body (DW, m)	Positive	2845	153.7531	2617.68	415.1449
Terrain rugged index (TRI)	Positive	0.526	0.00718	0.499	0.0156
Normalized difference vegetation index (NDVI)	Positive	0.2734	0.0062	0.2848	0.0151

We tested various potential anthropogenic variables to quantify the impact of human‐related threats on Chinese pangolins which influences their occurrence. Based on preliminary surveys conducted between September and October 2020, along with a review of existing literatures, we identified six major anthropogenic threats: vegetation destruction (VD) from logging and cutting; human‐caused forest fires; livestock grazing; fodder collection; foot and vehicular trails; and poaching activities, including the excavation of Chinese pangolin burrows using smoke to drive them out. Given the limited amount of literature detailing the extent of threats to the Chinese pangolins, we adapted a framework, based on previous studies (Barber‐Meyer et al. 2013; Pokharel et al. 2022; Thapa and Kelly 2017), incorporating necessary modification for quantifying the threats.

Along each 600 m transect, we collected quantifiable data on threats for the Human Disturbance Index (HDI) at every 100 m segment. This included factors such as VD, forest fires (F), livestock grazing and related indicators (L), human presence, foot and vehicle trails (H), and signs of pangolin poaching (K). Assigning equal weights (0.2) to each variable to minimize underestimation or overestimation of the influence of certain variables, we calculated human disturbance for each 100 m segment using the formula HDI = (VD × 0.2) + (F × 0.2) + (L × 0.2) + (H × 0.2) + (K × 0.2) and then averaged the HDI values for each study grid. We considered values up to 0.40 as an indication of low disturbance, 0.41 to 0.60 to signify moderate disturbance and 0.61 to 1.0 as representing high disturbance in the HDI. Due to the difficulty in detecting underground colonies of termites, we focused our efforts on recording above ground mounds of termites.

Throughout the survey, we georeferenced the majority of water bodies in the study grids. For those water bodies that we failed to locate during surveys, they were later digitized using Google Earth Imagery. The distance from the centroid of the study grid to the nearest water sources was calculated using Euclidean distance tool in ArcGIS 10.5. Due to simplicity of use and interpretation, NDVI is one of the widely adopted vegetation indices as a replacement for canopy cover (Tenreiro et al. 2021). We used NDVI derived from 30 m resolution Landsat 8 imagery captured during November 2020. We calculated the NDVI using the near infrared spectral reflectance (NIR) and visible (red) spectral reflectance (VIS) outlined by Wu et al. (2014). The values were extracted for each study grid's centroid point using ArcGIS 10.5 (Bista, Panthi, and Weiskopf 2018; Chatterjee and Basu 2018; Tenreiro et al. 2021). The topographic heterogeneity was assessed by calculating Terrain Ruggedness Index (TRI) introduced by Riley, DeGloria, and Elliot (1999). This evaluation was conducted using 30 m Shuttle Radar Topographic Mission (SRTM) Digital Elevation Model (DEM) data (https://srtm.csi.cgiar.org/) with the TRI value averaged per study grid (Thapa, Kelly, and Pradhan 2019). Additionally, habitat types including dominant *Shorea robusta* forests, mixed forests, riverine forests, grasslands, scrublands, settlement areas, barren land, wetlands, and agricultural land, based on their land cover patterns and habitat structures such as the Churia/Siwalik Hills and Terai were also quantified, as these factors significantly influence the distribution of the Chinese pangolin (Katuwal, Sharma, and Parajuli 2017; Shrestha et al. 2021; Suwal et al. 2020).

### 2.4 Data Analyses

The difference in the number of burrows observed and expected for the different habitat types were tested for the statistical significance by Chi‐square goodness‐of‐fit test. We utilized the Unmark package (version 1.4.1; Kellner et al. 2023) in R software v.4.4.1 (R Core Team 2023) to conduct a single‐season occupancy hierarchical model following the approach outlined by Fiske and Chandler (2011). This model estimates the maximum likelihood of observing a detection history, considering both detections and non‐detections at a site. Model selection and comparison were performed using the Akaike Information Criterion (AIC) value and to calculate their Akaike weights (Burnham, Anderson, and Burnham 2002). To begin, a detection history was produced, and the continuous variables were standardized by subtracting the mean from each observed value and dividing by the standard deviation and checked for multicollinearity using Spearman's rank correlation coefficient to avoid model overfitting. None of the covariates showed a strong correlation of (|0.7|) (Figure S1). In the case of categorical covariates, we performed a chi‐square test to examine the relationship between habitat type and structure. We found that both variables were not significantly correlated and hence included in the analysis. We employed a three‐step modeling approach to characterize the parameters of interest, as outlined by Karanth et al. (2011) and Srivathsa et al. (2018). Initially, we compared the standard occupancy model (Mackenzie and Royle 2005) with a model that accounted for correlated detections across spatial replicates (Kellner et al. 2023). This initial focus on the correlated detection model was based on the anticipation of spatial dependence in sign detection events across consecutive spatial replicates. After identifying the most suitable model, we then modeled the detection parameters, considering both a constant form and models that included individual covariates (Pokharel et al. 2022; Thapa, Kelly, and Pradhan 2019). During this step, the occupancy parameter remained in a fully parameterized form. In the final step, we modeled occupancy by setting the covariate structure for detection probability based on the top‐ranked model from the previous steps (Karanth et al. 2011; Srivathsa et al. 2018).

Models with ΔAIC < 2 were considered as competing models, and the final estimates of site use probability and detectability were calculated (Burnham, Anderson, and Burnham 2002). We computed *β* estimates of the covariates to understand the magnitude and direction (positive or negative) of their effects on the site use and detection probability. Finally, estimated probability from the study area was used to prepare a predicted map (Pokharel et al. 2022) using ArcGIS 10.5.

## 3 Results

### 3.1 Distribution of Chinese Pangolin Burrows

The survey covered a total length of 80.4 km using continuous transect walks in 134 grids. A total of 43 fresh burrows of Chinese pangolins, distributed across 20 study grids, were identified. This translates to a naive occupancy estimate of 0.128. We observed a heterogeneous distribution of fresh burrows across the study area. The *Shorea robusta* ‐dominated forest showed the highest burrow density, with 30 burrows recorded, exceeding the expected count of 20.9. In the Mixed Forest, 13 fresh burrows were identified, which is lesser than the expected count of 13.49. No burrows were found in riverine forests, grasslands, scrublands, settlements, barren land, wetlands, or agricultural areas, despite an expected count of 8.5 in these habitats (Table S1). There was a significant difference between the observed and expected counts of burrows (*χ* ^2^ = 12.391, *p* < 0.01) signifying the importance of *Shorea robusta* forests to Chinese pangolins.

In terms of the geographical distribution of fresh burrows, 65.1% (*n* = 28) of fresh burrows were located in the Terai region (flat lowlands), characterized by an average Terrain Rugged Index (TRI) of 0.47. The Churia Hills accounted for the remaining 34.9% (*n* = 15) of fresh burrows, with a TRI of 0.53. The detection of fresh burrows was influenced by the number of termite mounds (Table 2). The average HDI for the non‐detected study grids (mean ± SD = 0.78 ± 0.019) was significantly higher compared to detected grids (mean ± SD = 0.475 ± 0.04, *t* = 6.85, *p* < 0.0001).

**TABLE 2:** Models of burrow detection probability with site covariates of Chinese pangolins in Dharan Sub‐Metropolitan City.

Model	nPars	AIC	Delta AIC	AICwt	cumltvWt
*p*(Termite mounds) psi(.)	3	242.9442236	0	1	1
*p*(Habitat type) psi(.)	5	286.4169439	43.47272037	3.63E‐10	1
*p*(.) psi(.)	2	290.7618796	47.817656	4.14E‐11	1
*p*(Habitat structure) psi(.)	3	292.4080616	49.46383802	1.82E‐11	1
*p*(HDI) psi(.)	3	292.6522348	49.70801126	1.61E‐11	1
*p*(TRI) psi(.)	3	292.6818125	49.7375889	1.58E‐11	1
*p*(NDVI) psi(.)	3	292.7599496	49.81572603	1.52E‐11	1
*p*(DW) psi(.)	3	292.7618747	49.8176511	1.52E‐11	1

Abbreviations: DW, distance from nearest water body; HDI, human disturbance index; NDVI, normalized difference vegetation index; TRI, terrain ruggedness index.

### 3.2 Detection Probability of Chinese Pangolins

**FIGURE 2:**
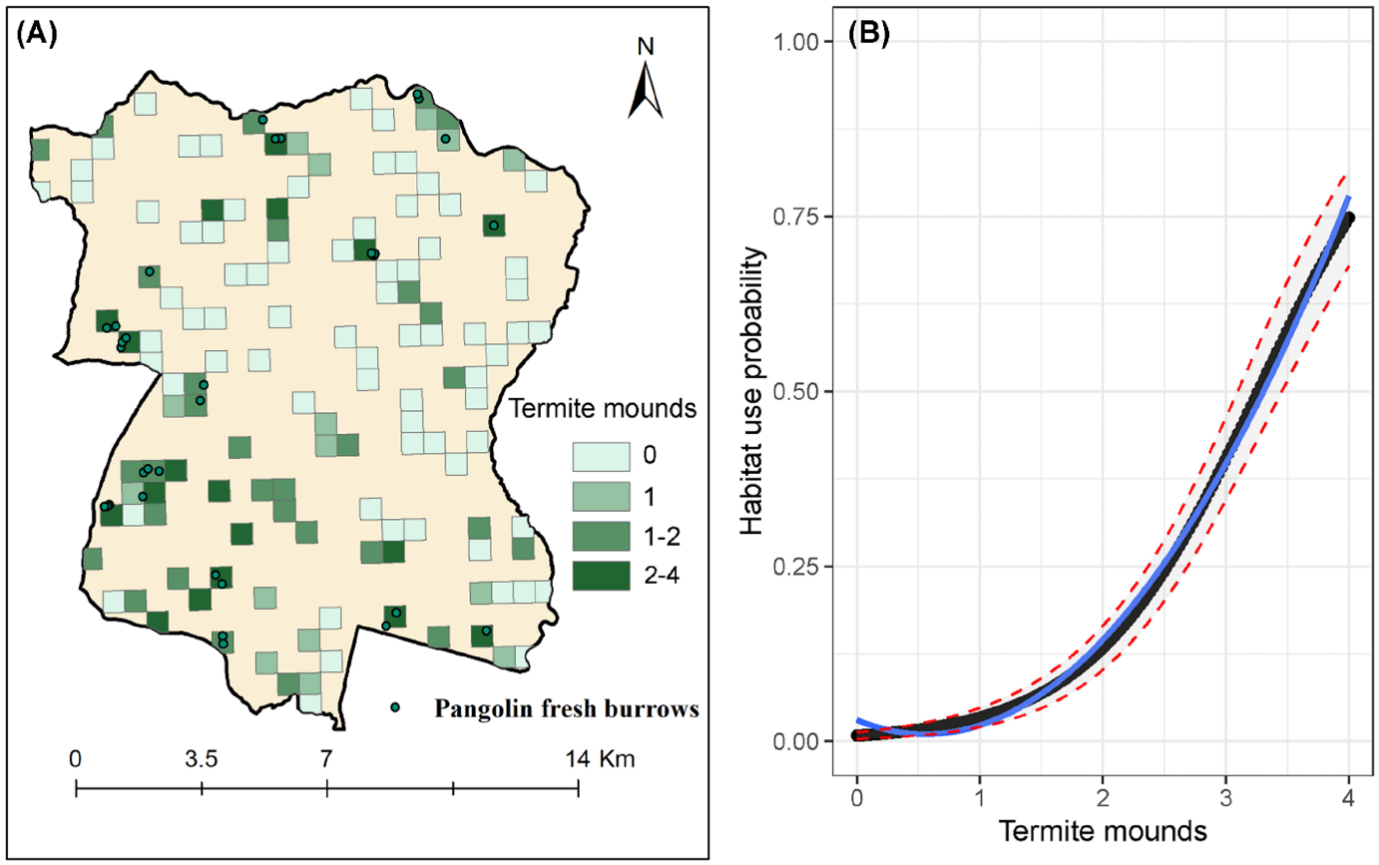
Relationship between the number of termite mounds and the detection probability of Chinese pangolin fresh burrows in Dharan Sub‐Metropolitan City. (A) Distribution of Chinese pangolin fresh burrows and termite mounds in the study grids; (B) a logistic regression line is shown in blue, with a gray shaded region bounded by red dashed lines indicating the 95% confidence interval of the detection probability.

We conducted eight regression models to analyze burrow detection probability, with one of them including the constant detection *p*(.). The model incorporating the termite mounds as covariates emerged as the top model (AICwt = 1) (Table 2, Table S1). The other models contributed negligibly compared to our top model. Notably, termite mounds significantly and positively influenced the detection probability of fresh pangolin burrows (*β*_Termite mounds_ = 1.48, 95% CI = 1.07 to 1.89) (Figure 2). The detection probability (*p* ± SE) of fresh pangolin burrows showed an average value of 0.095 ± 0.013 across the study grids. The range of detection probability among grids varied from a minimum of 0.0079 to a maximum of 0.75.

### 3.3 Occupancy of Chinese Pangolins

**FIGURE 3:**
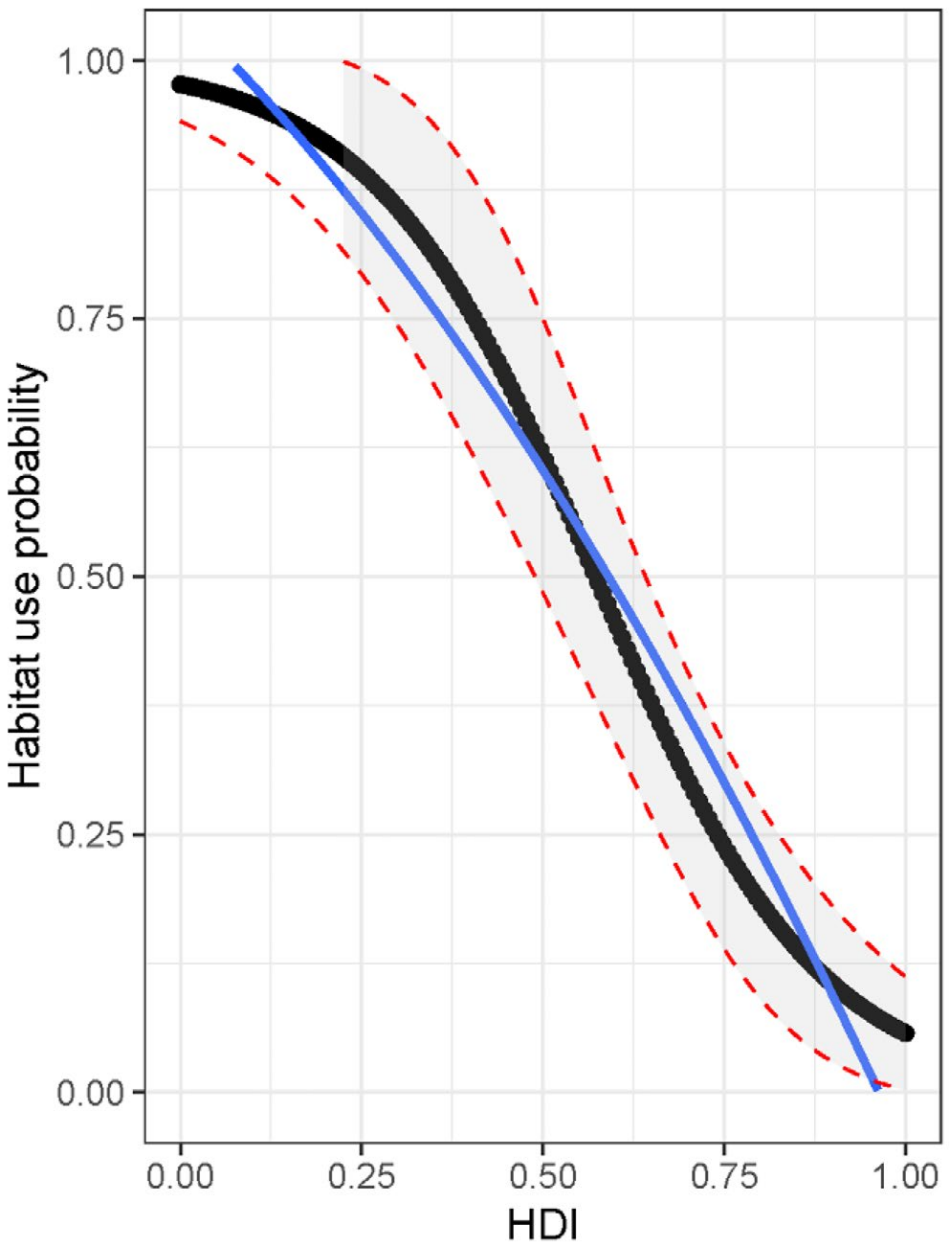
Relationship between the human disturbance index (HDI) and occupancy of Chinese pangolins in Dharan Sub‐Metropolitan City. The blue line represents regression line, and gray shaded region inside the red dashed lines represents the 95% confidence interval of the habitat occupancy.

We developed 25 regression models to assess habitat use occupancy of Chinese pangolins, each with the number of termite mounds as a fixed covariate for detectability (Table 3). Among these models, the one incorporating the HDI was found to be the top model for the occupancy (AIC = 231.96, AICwt = 0.309). Notably, the second to fifth models also demonstrated ΔAIC < 2, highlighting their competitive performance. However, we chose to prioritize the top model (AICwt = 0.309) for analysis because it consistently featured the inclusion of the HDI in all other models. To validate the top model, we conducted a Mackenzie–Bailey goodness‐of‐fit test through 1000 bootstrap iterations, yielding a significant result (*p* = 0.214). This provided a measure of how well the single‐season occupancy model aligns with the observed data and the models were accurate and the underlying assumptions were met. The analysis found that HDI has a significantly negative influence on the habitat occupancy (*β*_HDI_ = −6.555, 95% CI = −11.324 to −1.786) (Figure 3).

**TABLE 3:** Models assessing the factors affecting the likelihood of Chinese pangolin habitat use in Dharan Sub‐Metropolitan City through spatially replicated sign surveys.

Model	nPars	AIC	Delta AIC	AICwt	cumltvWt
*p*(Termite mounds) psi(HDI)	4	231.961	0	0.30953	0.30953
*p*(Termite mounds) psi(Habitat Structure + HDI)	5	232.326	0.36539	0.25785	0.56738
*p*(Termite mounds) psi(HDI + NDVI)	5	233.164	1.20339	0.16959	0.73697
*p*(Termite mounds) psi(HDI + TRI)	5	233.782	1.82091	0.12454	0.86151
*p*(Termite mounds) psi(HDI + DW)	5	233.934	1.9732	0.11541	0.97692
*p*(Termite mounds) psi(Termite mounds)	4	239.016	7.05484	0.00909	0.98601
~Termite mounds ~ Habitat Structure + Termite mounds	5	239.947	7.98605	0.00571	0.99172
~1 ~ Termite mounds + HDI	4	242.024	10.0634	0.00202	0.99374
*p*(Termite mounds) psi(.)	3	242.944	10.9833	0.00128	0.99502
*p*(Termite mounds) psi(Habitat Structure)	4	243.488	11.5267	0.00097	0.99599
*p*(Termite mounds) psi(HDI + Habitat Structure+NDVI)	6	243.712	11.7514	0.00087	0.99686
~Termite mounds ~ Habitat + Termite mounds	7	243.947	11.9863	0.00077	0.99763
*p*(Termite mounds) psi(NDVI)	4	244.049	12.0884	0.00073	0.99836
*p*(Termite mounds) psi(DW)	4	244.87	12.9089	0.00049	0.99885
*p*(Termite mounds) psi(TRI)	4	244.944	12.9833	0.00047	0.99932
*p*(Termite mounds) psi(HDI + Habitat Structure + NDVI+DW)	7	245.398	13.4367	0.00037	0.9997
*p*(Termite mounds) psi(Habitat)	6	246.899	14.938	0.00018	0.99987
*p*(Termite mounds) psi(Habitat + Habitat Structure)	7	247.539	15.5785	0.00013	1

Abbreviations: DW, distance from nearest water body; HDI, human disturbance index; NDVI, normalized difference vegetation index; TRI, terrain rugged index.

**FIGURE 4:**
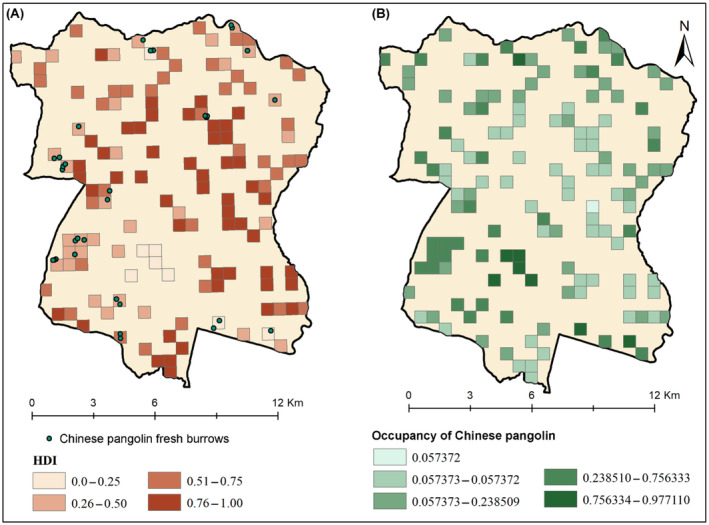
Chinese pangolin habitat use patterns in Dharan Sub‐Metropolitan City, eastern Nepal. (A) Naive estimate from presence‐versus‐absence approach also showing human disturbance index and (B) estimated probabilities of occupancy.

The estimated average habitat occupancy (Ψ ± SE) of fresh pangolin burrows in the study grids had a value of 0.316 ± 0.022. Similarly, the probability of occupancy (Ψ) values ranged from a minimum of 0.057 to a maximum of 0.977 (Figure 4) across the grids. Since neither the confidence intervals (CI) for our detection probability nor our occupancy values overlap with zero, our dataset is deemed suitable for analysis.

## 4 Discussion

This study represents an initial investigation into occupancy modeling of the Chinese pangolin within an urban setting in Nepal. Our research unveils key factors that impact the detection probability and occupancy of Chinese pangolins in the urban landscape of Dharan Sub‐Metropolitan City (Figure 4). It also provides crucial foundational insights into the factors affecting the distribution of Chinese pangolins in human‐dominated environments, particularly highlighting the impact of human disturbances. Chinese pangolins are known for their myrmecophagic behavior and dietary specialization on specific ants and termites (Lee et al. 2017; Tamang, Sharma, and Belant 2022; Wu et al. 2005). They exhibit foraging behavior positively influenced by termite mounds (Sharma, Rimal, et al. 2020, Sharma, Sharma, et al. 2020) and the proximity to ant and termite mounds or nests (Tamang, Sharma, and Belant 2022). This association likely explains why termite mounds significantly increased the detectability of Chinese pangolins in our study area. The population density and spatial utilization patterns of small mammals are determined by the availability and distribution of the key resources (Prevedello et al. 2013). Although the detection probability across the study area was relatively low (*p* ± SE, 0.095 ± 0.013), this can be attributed to the presence of termite mounds in both detected and non‐detected grids. During the field surveys, we observed pangolins primarily excavate soft topsoil, which is rich in ants and termites. The soft soil, characterized by moisture retention, creates a favorable environment for the growth and reproduction of ants and termites (Cornelius and Osbrink 2010).

The results support our hypothesis that anthropogenic activities negatively impact the occupancy of Chinese pangolin. The HDI emerged as the most influential factor negatively affecting the occupancy of Chinese pangolins in the study area. The average HDI across all study grids was notably higher than the average value for the grids, in which Chinese pangolin burrows were detected. Although the detected grids indicated an average HDI of 0.475, indicating the presence of moderate human disturbances, the occupancy of Chinese pangolins decreased as the HDI increased. The average estimated occupancy range was comparatively low due to the study area's location in a heavily human‐dominated region, where the local population had a heavy reliance on forest resources for necessities such as fodder and other essential materials.

Globally, the Chinese pangolin is the most heavily trafficked animals, sought after for its purported medicinal properties (Zhang et al. 2017) and as a culinary delicacy (Volpato et al. 2020). During our survey, we observed smoke near pangolin burrows, a common hunting method used by the people in the study area to drive the animals out into the open. The presence of smoked burrows suggested ongoing local hunting of the Chinese pangolins, although we were unable to confirm the precise motives for this activity. In Nepal, pangolins are also extensively trafficked, particularly in the central to eastern border regions (Bashyal et al. 2021; Suwal, Gurung, and Pei 2023). The primary drivers for hunting wild pangolins involve economic incentives, with hunters often living below the poverty line (Katuwal, Parajuli, and Sharma 2016; Sharma, Rimal, et al. 2020, Sharma, Sharma, et al. 2020).

Chinese pangolins, like many small native species with limited adaptability, experience adverse effects on their ecology and behavior due to anthropogenic disruptions in urban environments (Caspi et al. 2022; Schuttler et al. 2021). Similar to other small prey species such as brown bandicoots and squirrel gliders (Brady et al. 2011; FitzGibbon 2015), Chinese pangolins adapt their spatial behavior to cope with the urban stressors such as predator signals and human disturbances, which gradually decrease with increasing distance from urban areas. In moderately distant environments, subtle changes in activity patterns were more apparent among these animals throughout the night. Conversely, in more remote surroundings, small prey animals primarily cope with stressors by altering their habitat usage (Fardell et al. 2021; Schuttler et al. 2021). These findings highlight the variability in vigilance behavior as a response to stress, with some species avoiding it by modifying their spatial and temporal behaviors (Prevedello et al. 2013). Katuwal, Sharma, and Parajuli (2017) reported that Chinese pangolins actively avoid human disturbances; however, their study was conducted in rural areas of Nepal. In contrast, our study, conducted in an urban landscape, showed that the study grids (with detected burrows) experienced a moderate level of anthropogenic disturbances. Nevertheless, we observed a decrease in Chinese pangolin occupancy as the HDI increased, thus showing that human disturbance does impact their spatial activity. The manner in which Chinese pangolins cope with this environmental pressure, however, remains unclear.

### 4.1 Conservation Implications

Over the past decade, there has been a surge in research focusing on distribution modeling and factors influencing the habitat use of the Chinese pangolins (Katuwal, Sharma, and Parajuli 2017; Sharma, Rimal, et al. 2020, Sharma, Sharma, et al. 2020; Suwal et al. 2020; Tamang, Sharma, and Belant 2022). The Government of Nepal also prepared a 5‐year Pangolin Conservation Action Plan (2018–2022) and also has designated the species as protected wildlife under the NPWC Act 1973 (DNPWC 1973, 2019). Despite these efforts, essential baseline data on the ecology and behavior of the Chinese pangolins in Nepal is still lacking. The majority of Chinese pangolins' habitat in Nepal lies outside the protected areas (Suwal et al. 2020). Further, the country is undergoing rapid urbanization, resulting in the conversion of the Chinese pangolin's suitable habitat into agricultural and settlement areas (Gautam et al. 2003). This rapid shifting of land dynamics in the periphery of urban areas with extensive forest fragmentation for development projects is a growing concern (Devkota et al. 2023).

Our study focuses attention on the evolving landscape of Nepal and emphasizes the need to safeguard Chinese pangolins beyond protected areas from the escalating anthropogenic threats. The spatial distribution of Chinese pangolins in our study area is intricately linked to the abundance of termite mounds. To ensure the species' long‐term survival, it is crucial to conserve areas with suitable *Shorea robusta‐*dominated forest and mixed forest where termite mounds are abundant. The elevated anthropogenic activities within our study area, coupled with three documented cases of smoked fresh pangolin burrows along with other signs of hunting, heighten the risk of local extinction. Therefore, it is essential for local authorities and conservation agencies to closely monitor the primary habitat of Chinese pangolins, designate safe zones, and educate local communities about their ecological significance and vulnerability.

In this regard, we suggest that the predictor of Chinese pangolin presence in the study area, that is, termite mounds and the HDI for the occupancy, also should be applicable to other lowlands and the Churia range in Nepal. In this context, the rapid conversion of suitable Chinese pangolin habitats in favor of unmanaged urban landscape, is impacting the distribution of Chinese pangolins in Nepal. In addition, the dependency on forest areas is also increasing with the increase in the number of people living along the lowland forest periphery. This underscores the urgency of adopting conservation measures to mitigate the vulnerability of the Chinese pangolin across broader spatial scales.

## Author Contributions

**Asmit Subba:** conceptualization (equal), data curation (equal), formal analysis (equal), investigation (equal), methodology (equal), visualization (equal), writing – original draft (lead). **Ganesh Tamang:** methodology (equal), supervision (equal), validation (equal), writing – review and editing (equal). **Sony Lama:** data curation (equal), methodology (equal), writing – review and editing (equal). **Jash Hang Limbu:** investigation (equal), visualization (equal), writing – review and editing (equal). **Nabin Basnet:** data curation (equal), investigation (equal), writing – review and editing (equal). **Randall C. Kyes:** methodology (equal), visualization (equal), writing – review and editing (equal). **Laxman Khanal:** conceptualization (equal), methodology (equal), supervision (equal), validation (equal), writing – review and editing (equal).

## Conflicts of Interest

The authors declare no conflicts of interest.

## Supporting information


Data S1.

## Data Availability

The data used in this study have been submitted and are accessible from the DRYAD data repository from the following link: https://doi.org/10.5061/dryad.73n5tb34t.
