# Supplementary material for: Habitat Occupancy of the Critically Endangered Chinese Pangolin (Manis pentadactyla) Under Human Disturbance in an Urban Environment: Implications for Conservation
Source: Ecol Evol. 2024 Dec 15;14(12):e70726. doi: 10.1002/ece3.70726 (PMC11646938; doi:10.1002/ece3.70726)
Supplement: Supplementary file 1 — Data S1. [file ECE3-14-e70726-s001.docx]

Supplementary file 1

**Habitat Occupancy of the Critically Endangered Chinese Pangolin (*Manis pentadactyla)* under Human Disturbance in an Urban Environment: Implications for Conservation**

Asmit Subba^1,2^, Ganesh Tamang^3^, Sony Lama^4^, Jash Hang Limbu^5^, Nabin Basnet^6^, Randall C. Kyes^7^, Laxman Khanal^1^*

^1^Central Department of Zoology, Institute of Science and Technology, Tribhuvan University, Kathmandu 44618, Nepal

^2^Nature Conservation and Study Center, Kathmandu, Nepal

^3^Central Campus of Technology, Dharan, Nepal

^4^School of Ecology and Nature Conservation, Beijing Forestry University, China

^5^College of Fisheries and Life Science, Shanghai Ocean University, Shanghai, China

^6^Central Department of Botany, Institute of Science and Technology, Tribhuvan University, Kathmandu 44618, Nepal

^7^Departments of Psychology, Global Health, and Anthropology, Center for Global Field Study, and Washington National Primate Research Center, University of Washington, Seattle, WA 98195, USA

*** Correspondence:**

Laxman Khanal, Central Department of Zoology, Institute of Science and Technology, Tribhuvan University, Kathmandu 44618, Nepal, Email: [laxkhanal@gmail.com](mailto:laxkhanal@gmail.com)


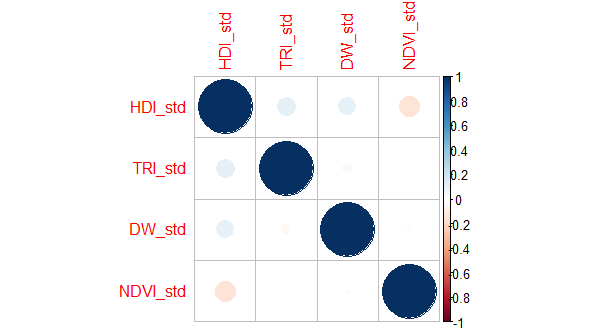


Figure S1: Pairwise correlation among the variables used in the analysis.

Table S1. Expected and observed Chinese pangolin burrows in different habitat types within the study area.

| **Habitat type** | **Area** | **Observed Burrows** | **Expected Burrows** |
| --- | --- | --- | --- |
| Agriculture | 4680 | 0 | 3.688416 |
| Human Settlement | 6120 | 0 | 4.823314 |
| Mixed Forest | 17120 | 13 | 13.492669 |
| Sal Forest | 26640 | 30 | 20.995601 |

Table S2. Detailed models assessing the factors affecting the likelihood of Chinese pangolin habitat use in the Dharan Sub-Metropolitan City through spatially replicated sign surveys.

| **Model** | **nPars** | **AIC** | **delta AIC** | **AICwt** | **cumltvWt** |
| --- | --- | --- | --- | --- | --- |
| p(Termite mounds) psi(HDI) | 4 | 231.961 | 0 | 0.30953 | 0.30953 |
| p(Termite mounds) psi(Habitat Structure + HDI) | 5 | 232.326 | 0.36539 | 0.25785 | 0.56738 |
| p(Termite mounds) psi(HDI + NDVI) | 5 | 233.164 | 1.20339 | 0.16959 | 0.73697 |
| p(Termite mounds) psi(HDI + TRI | 5 | 233.782 | 1.82091 | 0.12454 | 0.86151 |
| p(Termite mounds) psi(HDI + DW) | 5 | 233.934 | 1.9732 | 0.11541 | 0.97692 |
| p(Termite mounds) psi(Termite mounds) | 4 | 239.016 | 7.05484 | 0.00909 | 0.98601 |
| ~Termite mounds ~ Habitat Structure + Termite mounds) | 5 | 239.947 | 7.98605 | 0.00571 | 0.99172 |
| ~1 ~ Termite mounds + HDI) | 4 | 242.024 | 10.0634 | 0.00202 | 0.99374 |
| p(Termite mounds) psi(.) | 3 | 242.944 | 10.9833 | 0.00128 | 0.99502 |
| p(Termite mounds) psi(Habitat Structure) | 4 | 243.488 | 11.5267 | 0.00097 | 0.99599 |
| p(Termite mounds) psi(HDI +Habitat Structure+NDVI) | 6 | 243.712 | 11.7514 | 0.00087 | 0.99686 |
| ~Termite mounds ~ Habitat + Termite mounds) | 7 | 243.947 | 11.9863 | 0.00077 | 0.99763 |
| p(Termite mounds) psi(NDVI) | 4 | 244.049 | 12.0884 | 0.00073 | 0.99836 |
| p(Termite mounds) psi(DW) | 4 | 244.87 | 12.9089 | 0.00049 | 0.99885 |
| p(Termite mounds) psi(TRI) | 4 | 244.944 | 12.9833 | 0.00047 | 0.99932 |
| p(Termite mounds) psi(HDI +Habitat Structure+NDVI+DW) | 7 | 245.398 | 13.4367 | 0.00037 | 0.9997 |
| p(Termite mounds) psi(Habitat) | 6 | 246.899 | 14.938 | 0.00018 | 0.99987 |
| p(Termite mounds) psi(Habitat + Habitat Structure) | 7 | 247.539 | 15.5785 | 0.00013 | 1 |
| p(Habitat) psi(.) | 5 | 286.417 | 54.456 | 4.63E-13 | 1 |
| p(.) psi(.) | 2 | 290.762 | 58.8009 | 5.28E-14 | 1 |
| p(Habitat Structure) psi(.) | 3 | 292.408 | 60.4471 | 2.32E-14 | 1 |
| p(HDI) psi(.) | 3 | 292.652 | 60.6913 | 2.05E-14 | 1 |
| p(TRI) psi(.) | 3 | 292.682 | 60.7208 | 2.02E-14 | 1 |
| p(NDVI) psi(.) | 3 | 292.76 | 60.799 | 1.94E-14 | 1 |
| p(DW) psi(.) | 3 | 292.762 | 60.8009 | 1.94E-14 | 1 |
